# Hypoparathyroidism as the First Manifestation of Kearns-Sayre Syndrome: A Case Report

**Published:** 2013

**Authors:** Farah ASHRAFZADEH, Nosrat GHAEMI, Javad AKHONDIAN, Mehran BEIRAGHI TOOSI, Saghi ELMI

**Affiliations:** 1 Department of Pediatric Neurology, Ghaem Medical Center, Mashhahd University of Medical Sciences, Mashhad, Iran; 2 Department of Pediatric Endocrinology, Imam Reza Center, Mashhahd University of Medical Sciences, Mashhad, Iran; 3 Department of Pediatric Neurology, Medical Center, Mashhahd University of Medical Sciences, Mashhad, Iran; 4 Department of Pediatrics, Ghaem Medical Center, Mashhahd University of Medical Sciences, Mashhad, Iran

**Keywords:** Kearns-Sayre, Hypoparathyroidism, Ophthalmoplegia, Mitochondrial cytopathy

## Abstract

**Objective:**

Kearns-Sayre syndrome is a mitochondrial myopathy, which was first described by Tomas Kearn in 1958. Diagnostic symptoms include retinitis pigmentosa, chronic and progressive external ophthalmoplegia plus one or more of following factors: heart conduction system disorders, cerebellar ataxia, or cerebrospinal fluid (CSF) protein content above 100 mg/dL. The nature of this uncommon disease is yet to be clarified. In this paper, we report a case of Kearns-Sayre syndrome. According to the previous records, the first manifestation of Kearns- Sayre syndrome as hypoparathyroidism is uncommon and in this article, we report a case with this problem.

## Introduction

Kearns-Sayre syndrome is diagnosed with chronic and progressive external ophthalmoplegia (CPEO) and retinitis pigmentosa onset before the age of 20.

Clinical features are often associated with cerebellar ataxia, cardiac conduction block, elevated protein content of cerebrospinal fluid (CSF), and proximal myopathy. Children with Kearns-Sayre syndrome suffer from short stature and often endocrinopathies, including diabetes, parathyroid dysfunction, and Addison’s disease. Renal tubular acidosis (proximal and distal) exists in several cases, which is associated with renal failure and also bilateral hearing loss (1). 

Other symptoms may include:

1. Muscle weakness

a. Chronic and progressive loss of eye movements and ptosis

b. Dysphagia

skeletal muscle weakness (proximal more than distal), and exercise intolerance

2. CNS disorders

c. Cerebellar ataxia

d. Dementia, cognitive deficit, encephalopathy in acute presentation of lactic acidosis

3. Deafness

4. Night blindness

5. Heart disease (conduction disorders, congestive heart failure, and cardiomyopathy)

6. Endocrine disorders (diabetes, menstrual irregularities, delayed puberty, poor growth, seizures due to hypocalcemia because of parathyroid disease); so, screening for endocrinopathy is suggested by measuring serum electrolytes, glucose, calcium, magnesium, plasma cortisol level, and thyroid function (2-4).

**Patient report**

Our patient was a 15 year-old girl. She was produced by cesarean section. Birth weight was 2800 g. She had jaundice with phototherapy, which was improved without complication. Growth and development were normal until 3 years of age; with the onset of hair loss and hypocalcemia, then, she was referred to pediatric endocrinologist, and after the diagnosis of hypoparathyroidism was treated with calcium and calcitriol. After that, growth disorder and hearing loss were detected. After further investigation, it was proved that the patient had already had a hearing deficit. She was hospitalized at the age of 8 with polyuria and polydipsia due to DKA (diabetic ketoacidosis), and the diagnosis of type 1 diabetes was confirmed. Insulin therapy was started afterwards. At the age of 12, ptosis together with retinitis pigmentosa was detected in both eyes, which caused a decrease in visual acuity level and consequently led to difficulty in walking because of poor vision.

Intelligence quotient (IQ) level was normal, but because of the slight hearing loss, the ability to understand content decreased. By the age of 12, she was suffering from muscle weakness in all four limbs. 

In her family history, parents were relatives and her grandfather was suffering from diabetes mellitus (DM). Her siblings were in normal condition. 

**Physical examination**

**Fig 1:**
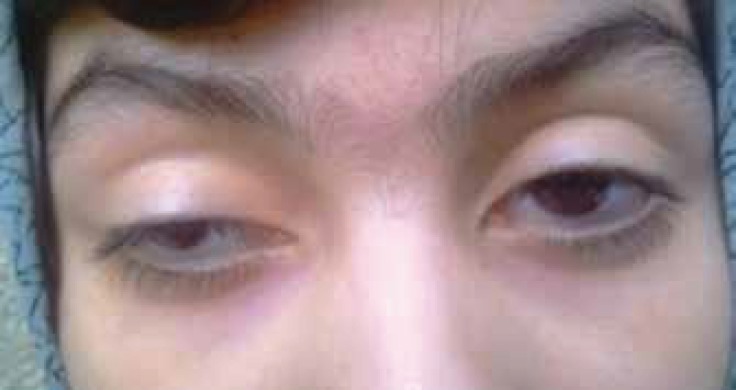
Bilateral ptosis

Blood pressure was about 120/80 mmHg; heart auscultation was normal; height was 140 cm; and weight was 38 kg. Force of muscles was estimated to be around 3/5 and DTRs (deep tendon reflexes) decreased, but the muscle tone was normal. Eye movements were slow and palsy of 3rd nerve, extraocular palsy, and bilateral ptosis were obvious (Figure 1).

 According to ophthalmology consultation, sight in both eyes was estimated around 2/10 with a maximum correction and bilateral severe dysfunction of eyelids and ptosis due to myopathy, probably Kearns-Sayre syndrome, CPEO, and bilateral retinal pigmentary changes with scar. In cases with severe eyelid impairment and ptosis, surgery may be needed.

Carotid doppler sonography was normal and abdominal ultrasound showed increased renal echogenicity, but DMSA scan was normal. Electromyography (EMG) and nerve conduction velocity (NCV) implicated in myopathic process with negative results in Jouly test. 

The assessments for autoimmune diseases, including anti TPO (thyroid peroxidase) antibody for thyroid assessment and anti TTG (tissue transglutaminase) antibody for celiac disease revealed no significant finding. Measurement of aldosterone and renin activity revealed normal results. In ACTH (adrenocorticotropin hormone) stimulation test, cortisol level was normal. Cell blood count (CBC) and Urin alysis were normal. Liver function tests, alkaline phosphatase, urea, and creatinine were within normal limits. Calcium was between 9.8 and 10.1 mg/dL, while she was under treatment for hypoparathyroidism. Hemoglobin A1C level was in the high range, which showed the improper control of diabetes. Genetic evaluation was not performed due to the lack of financial sources. 

**Fig 2:**
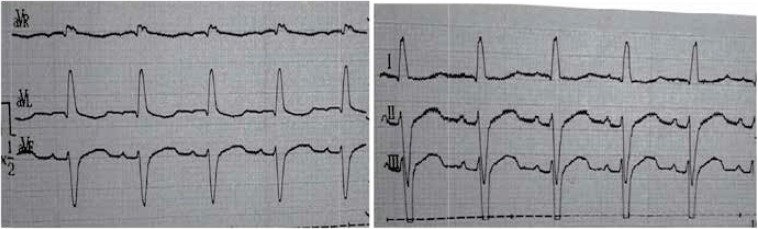
ECG of the patient that shows right hemi-block

In the Figure 2, the patient’s electrocardiography (ECG) is shown, and right hemi block and RV strain is obvious. Echocardiography showed cardiomyopathy. 

**Fig 3:**
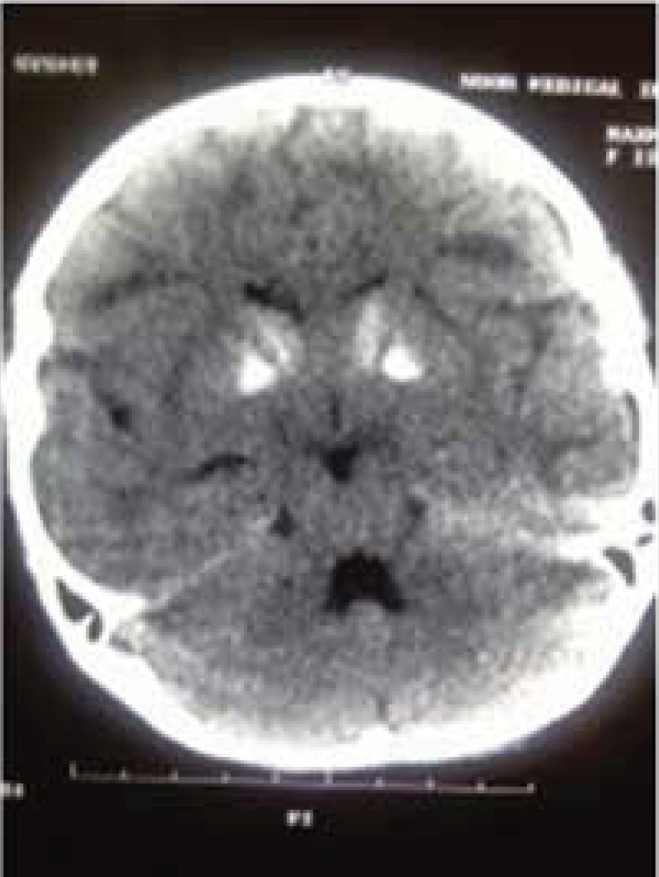
Brain CT scan shows bilateral calcification in basal ganglia, periventricular demyelination and mild dilatation of lateral ventricles

The brain CT scan showed calcification in basal ganglia, periventricular demyelinization, and mild dilatation of lateral ventricles (Figure 3).

**Fig 4:**
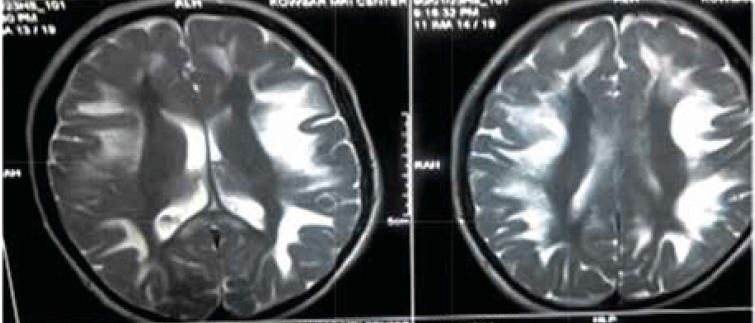
T2-weighted views of brain MRI shows high-intencity signals in periventricular white matter and midbrain

In T2-weighted and FLAIR (fluid-attenuated inversion recovery) views of brain MRI, abnormal brain signals from white matter and basal ganglia and midbrain were apparent, which are suggested for metabolic diseases or mitochondrial neuropathies (Figure 4).

Her speech was not eloquent. The patients’ audiometry showed sensorineural deafness. She could rely on herself for doing some of her daily routines. She was advised to undergo treatment with cardiac pacemaker for heart block, which can prevent sudden death. She was recommended to receive coenzyme Q10 (Ubiquinone) and vitamin supplements to support heart function and provide antioxidant protection. We encouraged her to follow her treatment better to control her DM 1 and hypoparathyroidism.

## Discussion

Inheritance pattern of Kearns-Sayre syndrome may be sporadic, autosomal dominant, or due to mutations in mtDNA (mitochondrial genome) that is inherited almost exclusively through the maternal lineage. Its prevalence is 1 to 3 cases per 100,000 people (5). The risk of maternal transmission has been estimated to be 1 in 24. The deletions vary in size and location. Amounts of deleted and wild-type mtDNA determine the phenotype. There is no sex or racial predilection. The onset is usually before 20 years of age (6). Bosbach et al.’s study in Germany showed that in patients with Kearns-Sayre syndrome, intelligence level is relatively normal and visual perception is impaired, and its progress indicates the impairment of prefrontal cortex (7). 

Cardiac complications are very common in patients with Kearns-Sayre syndrome. Berenberg et al. described most common cardiac features. In their study, the most common causes of death were cardiomyopathy and sudden cardiac death (8). Right Ventricle Strain and right hemi-block was evident in our patient’s ECG. In some patients, mitral or tricuspid valve prolapse was seen as well. AV block incidence is high in Kearns-Sayre syndrome, and most of the patients need pacemakers. Welzingin et al. in 2008 described Kearns-Sayre as a mitochondrial encephalopathy with increased level of CSF protein content (9). Berio et al. (10) found out the lack of growth hormone in Kearns-Sayre syndrome and Schmiedel et al. described myopia, nystagmus vestibular disorders, myopathy, nystagmus, and encephalopathy (11). Chu et al. described the brain signs in brain MRI as subcortical white matter involvement and other brain structures, such as brain stem, globus pallidus, and thalamus. (12) In our patient, calcifications in basal ganglia, periventricular demyelinization, and mild dilatation of lateral ventricles were obvious. Altunbaşak et al. in 1998 described cardiac involvement as the key symptom of disease, which may lead to death (13). Chawla et al. noted prolonged PR or QT intervals and sinus dysrhythmia (14). Muscle biopsy may reveal ragged red fibers and muscle histochemistry can determine deficiency of cytochrome c oxidase (16). Histological evaluation was not performed due to patient’s disagreement. ACC/AHA guideline in 2002 recommended pacemaker placement in neuromuscular diseases like Kearns-Sayre syndrome, even in symptom free patients (15). Also, folinic acid can cause clinical and radiological improvement, especially in leukoencephalopathy (16).

## Notes

**In conclusion, **Kearns-Sayre syndrome is a mitochondrial disease with mortal cardiac disorders before 20 years old and visual disturbance associated with endocrinopathy and myopathy. According to our report, it is important that each youth with endocrinopathy to be suspected of having mitochondrial disorders.

## References

[1] Ashizawa T, Subramony SH (2001). What is Kearns-Sayer syndrome after all?. Arch Neurol.

[2] Barragan-Campos HM, Vallee JN, Lo D, Barrera-Ramirez CF, Argote-Greene M, Sanchez-Guerrero J (2005). Brain magnetic resonance imaging findings in patients with mitochondrial cytopathies. Arch Neurol.

[3] Amemiya S, Hamamoto M, Goto Y, Komaki H, Nishino I, Nonaka I ( 2000). Psychosis and progressive dementia: presenting features of a mitochondriopathy. Neurology.

[4] Katsanos KH, Pappas CJ, Patsouras D, Michalis LK, Kitsios G, Elisaf M (2002). Alarming atrioventricular block and mitral valve prolapse in the Kearns-Sayer syndrome. Int J Cardiol.

[5] Tiranti V, Viscomi C, Hildebrandt T, Di Meo I, Mineri R, Tiveron C (2009). Loss of ETHE1, a mitochondrial dioxygenase, causes fatal sulfide toxicity in ethylmalonic encephalopathy. Nat Med.

[6] Chinnery PF, DiMauro S, Shanske S, Schon EA, Zeviani M, Mariotti C (2004). Risk of developing a mitochondrial DNA deletion disorder. Lancet.

[7] Bosbach S, Kornblum C, Schröder R, Wagner M (2003). Executive and visuospatial deficits in patients with chronic progressive external ophthalmoplegia and Kearns-Sayer syndrome. Brain.

[8] Berenberg RA, Pellock JM, DiMauro S, Schotland DL, Bonilla E, Eastwood A (1977). Lumping or splitting? “Ophthalmoplegia-plus” or Kearns-Sayer syndrome?. Ann Neurol.

[9] Welzing L, von Kleist-Retzow JC, Kribs A, Eifinger F, Huenseler C, Sreeram N (2009). Rapid development of life threatening complete atrioventricular block in Kearns- Sayer syndrome. Eur J Pediatr.

[10] Berio A, Piazzi A (2000). Kearns-Sayer syndrome with GH deficiency. Pediatr Med Chir.

[11] Schmiedel J, Jackson S, Schäfer J, Reichmann H ( 2003). Mitochondrial cytopathies. J Neurol.

[12] Chu BC, Terae S, Takahashi C, Kikuchi Y, Miyasaka K, Abe S (1999). MRI of the brain in the Kearns-Sayer syndrome: report of four cases and a review. Neuroradiology.

[13] Altunbaşak S, Bingöl G, Ozbarlas N, Akçören Z, Hergüner O (1998). Kearns-Sayer syndrome. A case report. Turk J Pediatr.

[14] Chawla S, Coku J, Forbes T, Kannan S (2008). Kearns-Sayer syndrome presenting as complete heart block. Pediatr Cardiol.

[15] Gregoratos G, Abrams J, Epstein AE, Freedman RA, Hayes DL, Hlatky MA (2002). ACC/AHA/NASPE 2002 guideline update for implantation of cardiac pacemakers and antiarrhythmia devices: summary article: a report of the American College of Cardiology/American Heart Association Task Force on Practice Guidelines (ACC/AHA/NASPE Committee to Update the 1998 Pacemaker Guidelines). Circulation.

[16] Basu AP, Posner E, McFarland R, Turnbull DM (Feb 4, 2010). Kearnsayre syndrome. http://emedicine.medscape.com/article/950897.

